# Flavanone Intake and Cardiometabolic Outcomes: A Synthesis of Meta-Analytic Evidence from Cohort and Clinical Studies

**DOI:** 10.3390/epidemiologia7040108

**Published:** 2026-08-12

**Authors:** Saeid Doaei, Mercedes Gil-Lespinard, Zehra Elban, Maryam Gholamalizadeh, Enrique Almanza-Aguilera, Raul Zamora-Ros

**Affiliations:** 1 Unit of Nutrition and Cancer, Cancer Epidemiology Research Programme, Catalan Institute of Oncology (ICO), Bellvitge Biomedical Research Institute (IDIBELL), L’Hospitalet de Llobregat, 08908 Barcelona, Spain; sdoaei@idibell.cat (S.D.); gilmercedes21@gmail.com (M.G.-L.); zehra.elbnn@gmail.com (Z.E.); mgholamalizadeh@idibell.cat (M.G.); ealmanzaa@outlook.com (E.A.-A.); 2 Department of Community Nutrition, School of Nutrition and Food Sciences, Shahid Beheshti University of Medical Sciences, Tehran 19816-19573, Iran; 3 Division of Food and Nutrition Science, Department of Life Sciences, Chalmers University of Technology, 41296 Gothenburg, Sweden

**Keywords:** flavanones, flavonoids, cardiometabolic health, cardiovascular diseases, stroke, obesity

## Abstract

Flavanones, a subclass of flavonoids predominantly found in citrus fruits, have attracted growing interest for their potential role in cardiometabolic health. While evidence linking overall flavonoid intake to cardiometabolic benefits is relatively consistent, flavanones have received less attention, and available findings remain scattered across heterogeneous outcomes and study designs. This review synthesizes meta-analytic evidence from prospective cohort and intervention studies to provide a comprehensive assessment of the relationship between flavanone intake and cardiometabolic health. A comprehensive literature search was performed to identify published meta-analyses examining flavanone intake in relation to cardiometabolic outcomes and related biomarkers. Twenty-five eligible meta-analyses, including 12 meta-analyses of prospective cohort studies and 13 meta-analyses of randomized controlled trials, were included. Evidence from prospective cohort studies showed that higher flavanone intake was associated with a lower risk of cardiovascular disease (relative risk reductions ranging from 12% to 22%) and stroke (relative risk reductions ranging from 12% to 26%). Intervention studies demonstrated favorable effects on total cholesterol, LDL cholesterol, systolic blood pressure, fasting glucose, and C-reactive protein concentrations, although findings for anthropometric outcomes were inconsistent. Overall, the current evidence supports a potential protective role of flavanone intake in cardiometabolic health. However, these findings should be interpreted with caution given the heterogeneity of studies and outcome measures, as well as methodological limitations within the underlying meta-analyses.

## 1. Introduction

Cardiometabolic diseases, including cardiovascular diseases (CVDs) and metabolic disorders are the leading cause of mortality worldwide [1]. Recent estimates indicate that CVD alone affected more than 600 million individuals in 2021 [2]. The increasing burden is largely driven by metabolic risk factors such as hypertension, obesity, dyslipidemia, and impaired glucose regulation, reflecting the ongoing global rise in metabolic disorders [3]. Environmental and lifestyle factors, including diet quality and intake of bioactive dietary components, are increasingly recognized as major contributors to cardiometabolic risk [4].

(Poly)phenols are bioactive compounds present in plant foods that exhibit antioxidant properties [5]. These compounds may influence gene expression through epigenetic mechanisms and have been associated with the prevention of cardiometabolic diseases [6,7]. Particularly, citrus (poly)phenols exhibit anti-inflammatory and antioxidant properties in experimental studies [8,9]. They have been linked to a reduced risk of CVD and intermediate risk factors, such as dyslipidemia and obesity [10,11]. Associations have also been reported with type 2 diabetes and certain cancers [12,13].

Flavanones are a subclass of flavonoids, a major class of dietary (poly)phenols [14], that are predominantly found in citrus fruits and include hesperidin, narirutin, naringin, eriocitrin, and their corresponding aglycones (hesperetin, naringenin, and eriodictyol) [15]. Oranges and orange juice are the major dietary sources of hesperidin and narirutin, whereas grapefruits are particularly rich in naringin [16,17]. Structurally, flavanones are characterized by a flavan nucleus composed of two aromatic rings linked by a dihydropyrone ring [10]. Flavanones exist mainly as glycosides, and flavanone content varies according to citrus species, cultivar, and processing conditions [15]. Dietary intake estimates suggest that the intake of flavanones varies widely across populations, with higher intakes reported in Mediterranean regions (33.2 mg/d expressed as aglycones) compared to Northern European populations (23.6 mg/d expressed as aglycones) [18].

Although flavanones are considered potentially protective dietary compounds, findings from cohort and intervention studies remain heterogeneous across cardiometabolic outcomes and study designs [19]. To date, no review has specifically summarized meta-analytic evidence on flavanones in relation to cardiometabolic outcomes. This review aimed to synthesize meta-analytic evidence from prospective cohort and interventional studies on the association between dietary flavanone intake and cardiometabolic outcomes and related biomarkers. While evidence from cohorts primarily examines relationships with disease risk, findings from interventional studies provide complementary insight into potential biological mechanisms through changes in cardiometabolic biomarkers.

## 2. Materials and Methods

This review aimed to summarize the available evidence from published meta-analyses assessing the association between flavanone intake and cardiometabolic outcomes.

A comprehensive literature search was conducted in PubMed, Web of Science, and Google Scholar databases to identify systematic reviews and meta-analyses evaluating the associations between flavanones and cardiometabolic outcomes. The literature search was updated to July 2026 to identify the most recent eligible meta-analyses. The search combined terms related to flavanones (e.g., flavanones, hesperidin, hesperetin, naringin, naringenin, citrus fruits, orange juice, and grapefruit) with terms related to cardiometabolic outcomes (e.g., cardiovascular disease, coronary heart disease, stroke, type 2 diabetes, obesity, blood pressure, blood lipids, glucose metabolism, inflammatory biomarkers, and anthropometric measures). Database-specific filters for systematic reviews and meta-analyses were applied where appropriate (Supplementary Materials File S1). In addition, the reference lists of all eligible articles were manually screened to identify any additional relevant studies.

### 2.1. Eligibility Criteria

The review was conducted according to the Preferred Reporting Items for Systematic Reviews and Meta-Analyses (PRISMA 2020) statement (Supplementary Materials File S2). Eligible studies were systematic reviews with meta-analyses of prospective cohort studies or randomized controlled trials conducted in human adults. Reviews evaluating dietary flavanones, flavanone-rich citrus fruits or citrus juices, or specific flavanones (e.g., hesperidin, hesperetin, naringin, and naringenin) in relation to cardiometabolic diseases or related risk factors were included.

Studies were excluded if they were narrative reviews, editorials, conference abstracts, or protocols; did not include a quantitative meta-analysis; were based exclusively on animal, in vitro, or mechanistic studies; evaluated non-cardiometabolic outcomes (e.g., neurological, rheumatological, hepatic, or pharmaceutical outcomes); investigated compounds outside the scope of flavanones without reporting flavanone-specific results; or were duplicate publications, in which case the most complete or most recent publication was retained.

### 2.2. Study Selection

**Figure 1:**
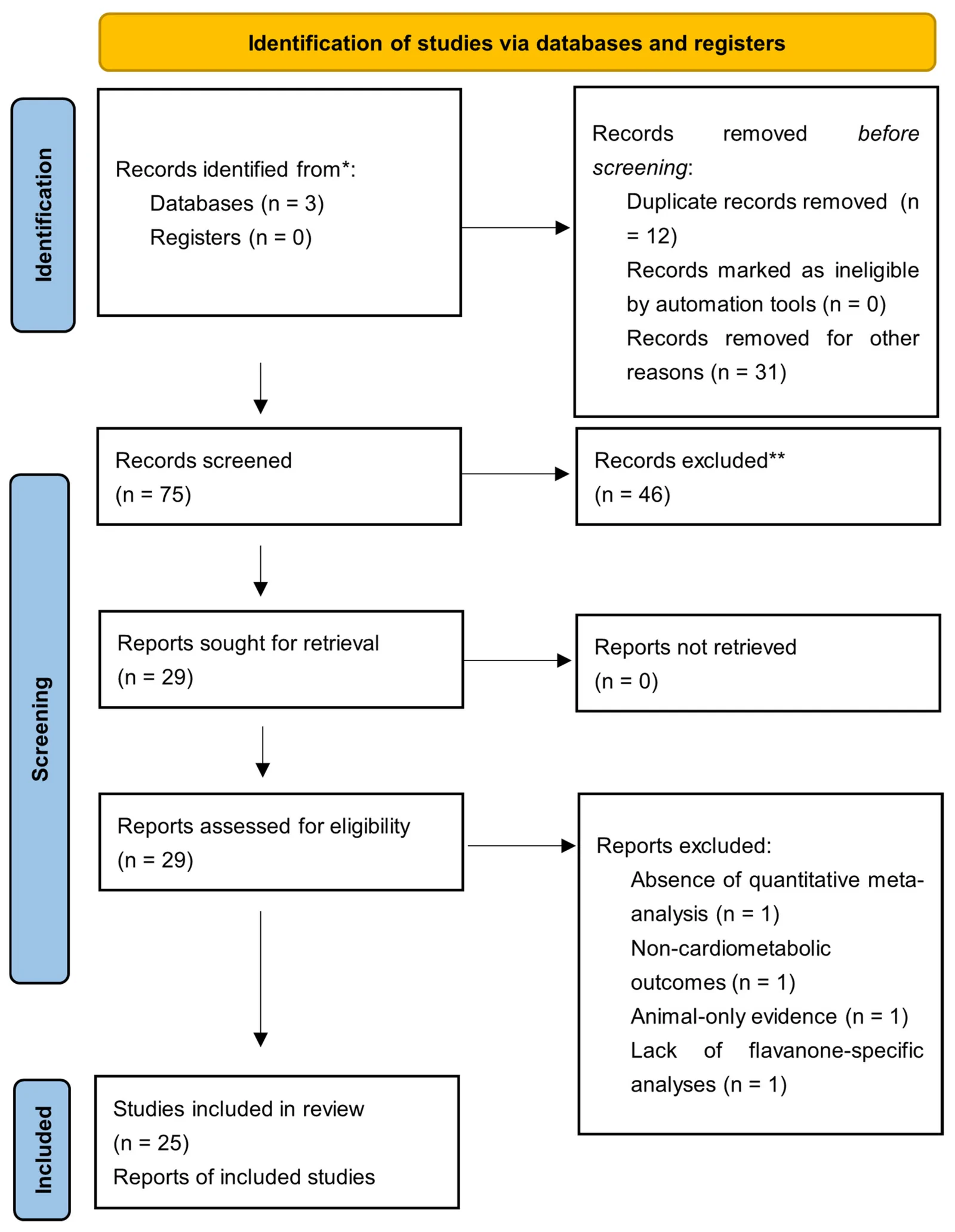
PRISMA 2020 flow diagram [20] for the umbrella review of meta-analyses on flavanone intake and cardiometabolic outcomes. * Records identified from the three databases searched: PubMed, Web of Science, and Google Scholar. ** Records excluded during title/abstract screening because they were narrative reviews, preclinical studies, non-cardiometabolic reviews, network meta-analyses, or did not evaluate flavanone-related outcomes.

The literature search was rerun using the final search strategy to generate the PRISMA flow diagram and ensure reproducibility of the study selection process (Figure 1).

The database search identified 118 records, of which 12 duplicate records and 31 records were removed before screening, leaving 75 records for screening. Of these, 46 records were excluded because they were narrative reviews, preclinical studies, non-cardiometabolic reviews, or network meta-analyses, or they did not evaluate flavanone-related intake or flavanone-based interventions. Twenty-nine full-text articles were assessed for eligibility, and four were excluded because they did not meet the predefined inclusion criteria (e.g., absence of quantitative meta-analysis, non-cardiometabolic outcomes, animal-only evidence, or lack of flavanone-specific analyses). Ultimately, 25 meta-analyses were included in this overview.

The review included 25 meta-analyses, comprising meta-analyses of prospective cohort studies (n = 12) and randomized controlled trials (n = 13). Given the aim of a two-layer synthesis integrating evidence from meta-analyses of both prospective cohort and intervention studies, and the substantial heterogeneity in intake definitions (flavanones, citrus fruits, and citrus-derived compounds) and cardiometabolic outcomes, a formal umbrella review was not undertaken. Instead, a targeted narrative synthesis approach was used to allow a more flexible and concept-driven integration of the available evidence.

ChatGPT (GPT-5.6 Luna; OpenAI, San Francisco, CA, USA) was used to assist with language editing, grammar correction, and improving the clarity and readability of the manuscript. The authors reviewed and verified all AI-assisted content and take full responsibility for the final content of the manuscript.

## 3. Results

### 3.1. Cohort Studies on Flavanones and Cardiometabolic Health

Table 1 summarizes meta-analyses of prospective cohort studies evaluating flavanone intake and cardiometabolic outcomes. Overall, the included meta-analyses comprised adult populations from Europe, the United States, and Asian countries, particularly Japan and China. The analyses evaluated flavanone intake from dietary sources, mainly citrus fruits, citrus juices, and total flavanone intake, in relation to CVD, CHD, stroke, and type 2 diabetes incidence and mortality outcomes. Across the included meta-analyses, several hundred thousand participants and thousands of cardiometabolic events were analyzed.

Seven meta-analyses have assessed the association between flavanones or flavanone-rich citrus sources and the risk of CVD incidence or mortality [21,22,23,24,25,26,27]. Micek et al. [21] included studies in Western populations and reported a 12% lower risk of CVD (incidence and mortality) for the highest versus lowest flavanone intake categories [RR: 0.88 (0.79; 0.98)]. Likewise, Aune et al. [22] reported an inverse association between higher citrus fruit intake and CVD risk [RR: 0.78 (0.66–0.92)], but not for citrus juice or dose–response analyses. For CVD mortality, three meta-analyses reported a 16% [RR: 0.84 (0.73–0.96)] [24], 14% [0.86 (0.76–0.97)] [23], and 12% [0.88 (0.82–0.96)] [25] lower risk for the highest compared with the lowest category of total flavanone intake. In contrast, in a fourth meta-analysis, no statistically significant results were observed for the intake of citrus fruits and CVD mortality [27].

For coronary heart disease (CHD), only one meta-analysis reported no significant association with total flavanone intake [21]. In contrast, regarding the intake of citrus fruits, Zurbau et al. [27] reported a 9% lower risk of CHD incidence [RR: 0.91 (0.85–0.98)] and mortality [RR: 0.91 (0.85–0.96)] associated with higher citrus fruit intake.

Four meta-analyses examined the association of flavanone or citrus fruit consumption and the risk of stroke (incidence or mortality) [21,22,27,28]. Micek et al. [21] and Li et al. [28] reported a 14% and 15% lower risk of stroke, respectively, associated with higher flavanone intake. Regarding citrus fruit intake, Aune et al. [22] reported inverse associations between citrus fruit intake and stroke risk both for high compared with low intake [RR: 0.74 (0.65; 0.84)] and in dose–response analyses per 100 g/d [RR: 0.78 (0.69; 0.90)]. They also observed inverse associations for ischemic stroke, with lower risk for higher intake of citrus fruits and citrus juices. In dose–response analyses, each 100 g/day increase in intake was associated with a relative risk of 0.87 (0.79–0.95) and 0.87 (0.80–0.96), respectively. Zurbau et al. [27] also observed inverse associations between higher citrus fruit intake and both total stroke incidence [RR: 0.88 (0.82; 0.94)] and stroke-related mortality [RR: 0.90 (0.86; 0.95)] in a similar population.

For type 2 diabetes mellitus, four meta-analyses have assessed the relationship between higher intakes of total flavanone [29,30,31] or citrus fruit [32] and type 2 diabetes risk, but no significant association was observed.

Overall, prospective cohort evidence suggests an inverse association between flavanone intake and overall CVD risk, including CHD and stroke, whereas no significant association has been consistently observed for type 2 diabetes risk. However, most meta-analyses did not stratify results by sex or ethnicity, limiting the assessment of potential subgroup differences.

**Table 1:** Summary of meta-analyses of cohort studies assessing the association between intake of flavanones and risk of incidence or mortality of different cardiometabolic outcomes.

Author, Year (Ref)	Studies with Flavanones (n)	Region	Age (y)	Outcome	Total Participants (n)	Cases (n)	Intake	Heterogeneity (%)	RR (95% CI)
Total Cardiovascular Diseases (CVD)									
Micek et al., 2021 [21]	8	Australia, Finland, France, Italy, Spain, UK, USA	≥18	CVD incidence and mortality	254,063	11,959	High vs. low flavanone intake	63.4	0.88 (0.79; 0.98)
Aune et al., 2017 [22]	6	Japan, Norway, Spain, UK, USA	35 to 80	CVD incidence and mortality	245,742	11,093	High vs. low citrus fruit intake	72.3	0.78 (0.66; 0.92)
	8	Australia, China, Japan, Norway, Spain, UK, USA			249,490	12,107	Per 100 g/d citrus fruit intake	65.8	0.92 (0.84; 1.00)
	2	UK, USA	30 to 75		140,246	4178	High vs. low citrus fruit juice intake	48.3	0.88 (0.53; 1.46)
			35 to 75		140,093	4150	Per 100 g/d citrus fruit juice intake	6.9	0.98 (0.95; 1.02)
Zurbau et al., 2020 [27]	6	Norway, UK, USA	30 to 80	CVD incidence	222,525	6220	High vs. low citrus fruit intake	33.0	0.88 (0.80; 0.96)
Grosso et al., 2017 [24]	10	Australia, Finland, Italy, Spain, USA	29 to ≥75	CVD mortality	248,069	7419	High vs. low flavanone intake	34.0	0.84 (0.73; 0.96)
Kim and Je, 2017 [23]	4	Australia, Spain, USA	29 to ≥75	CVD mortality	147,326	5091	High vs. low flavanone intake	31.2	0.86 (0.76; 0.97)
Rienks et al., 2017 [26]	1	Denmark	50 to 64	CVD mortality	786	393	High vs. low urine flavanone concentration	-	0.68 (0.41; 1.12)
							High vs. low urine hesperetin concentration	-	0.72 (0.43; 1.18)
							High vs. low urine naringenin concentration	-	0.63 (0.38; 1.02)
Wang et al., 2014 [25]	4	Finland, USA	39 to 69	CVD mortality	144,962	6829	High vs. low flavanone intake	0.0	0.88 (0.82; 0.96)
Zurbau et al., 2020 [27]	3	Norway, UK, USA	35 to 69	CVD mortality	74,716	7197	High vs. low citrus fruit intake	62.0	0.95 (0.90; 1.02)
Stroke									
Aune et al., 2017 [22]	8	Finland, Japan, Netherlands, Norway, Spain, Sweden, UK	35 to 83	Total stroke incidence and mortality	237,996	8751	High vs. low citrus fruit intake	53.6	0.74 (0.65; 0.84)
	9	China, Finland, Japan, Netherlands, Norway, Spain, Sweden, UK			240,441	9173	Per 100 g/d citrus fruit intake	63.6	0.78 (0.69; 0.90)
	2	UK, USA	35 to 69		64,947	617	High vs. low citrus fruit juice intake	0.0	0.90 (0.74; 1.10)
							Per 100 g/d citrus fruit juice intake		0.89 (0.72; 1.10)
Li et al., 2022 [28]	6	Finland, France, USA	30 to 84	Total Stroke incidence and mortality	254,123	5072	High vs. low flavanone intake	0.0	0.85 (0.78; 0.93)
Micek et al., 2021 [21]	6	Finland, France, UK, USA	≥15	Total Stroke incidence and mortality	225,158	5393	High vs. low flavanone intake	0.0	0.86 (0.77; 0.96)
Zurbau et al., 2020 [27]	8	Finland, Japan, Netherlands, Spain, Sweden, USA	35 to 69	Total Stroke incidence	225,613	7142	High vs. low citrus fruit intake	51.0	0.88 (0.82; 0.94)
Zurbau et al., 2020 [27]	4	China, Japan, Norway, UK	35 to 69	Total Stroke mortality	145,204	3869	High vs. low citrus fruit intake	82.0	0.90 (0.86; 0.95)
Aune et al., 2017 [22]	7	Denmark, Finland, Japan, Sweden, USA	34 to 83	Ischemic stroke incidence and mortality	347,947	6336	High vs. low citrus fruit intake	66.6	0.78 (0.66; 0.92)
	6	Denmark, Finland, Japan, Sweden, USA			278,325	5393	Per 100 g/d citrus fruit intake	52.5	0.87 (0.79; 0.95)
	1	USA	40 to 75		38,683	204	High vs. low citrus fruit juice intake	0.0	0.65 (0.51; 0.84)
							Per 100 g/d citrus fruit juice intake		0.87 (0.80; 0.96)
Aune et al., 2017 [22]	3	Finland, Japan, Sweden	40 to 83	Hemorrhagic stroke incidence and mortality	89,576	1193	High vs. low citrus fruit intake	28.2	0.74 (0.55; 1.01)
							Per 100 g/d citrus fruit intake	32.2	0.79 (0.59; 1.06)
Coronary Heart Disease (CHD)									
Micek et al., 2021 [21]	5	Finland, France, UK, USA	≥15	CHD incidence and mortality	170,264	9303	High vs. low flavanone intake	50.0	0.96 (0.84; 1.09)
Zurbau et al., 2020 [27]	10	China, Denmark, France, Germany, Italy, Japan, Northern Ireland, Spain, USA	20 to 80	CHD incidence	364,978	8333	High vs. low citrus fruit intake	0.0	0.91 (0.85; 0.98)
Zurbau et al., 2020 [27]	6	China, Japan, Norway, UK, USA	≥35	CHD mortality	180,574	5309	High vs. low citrus fruit intake	0.0	0.91 (0.85; 0.96)
Type 2 Diabetes (T2D)									
Guo et al., 2019 [30]	4	Europe, Poland, Spain, USA	36 to 67	T2D incidence	225,388	14,110	High vs. low flavanone intake	46.1	1.01 (0.93; 1.10)
Xu et al., 2018 [29]	7	Europe, Finland, Poland, Spain, USA	26 to ≥45	T2D incidence	302,137	19,427	High vs. low flavanone intake	44.0	1.02 (0.94; 1.10)
Jia et al., 2016 [32]	6	China, Europe, USA	24 to ≥45	T2D incidence	304,391	16,102	High vs. low citrus fruit intake	0.0	1.02 (0.96; 1.08)
Rienks et al., 2018 [31]	7	Europe, Italy, Poland, USA	36 to 66	T2D incidence	245,182	24,737	High vs. low flavanone intake	46.1	1.01 (0.93; 1.10)

CHD, coronary heart disease; CI, confidence interval; CVD, cardiovascular disease; RR, relative risk; T2D, type 2 diabetes; USA, United States of America; UK, United Kingdom.

### 3.2. Randomized Controlled Trials on Flavanones and Cardiometabolic Health

The intervention meta-analyses evaluated two distinct intervention models: flavanone-rich foods and beverages (primarily orange juice, grapefruit juice, and citrus fruits) and purified flavanone supplements, predominantly hesperidin. Overall, favorable effects on several cardiometabolic biomarkers were reported across both intervention models; however, the evidence was more extensive for purified hesperidin supplementation than for dietary flavanone sources. Table 2 summarizes meta-analyses of randomized controlled trials (RCTs) assessing the effect of different types of flavanone supplementation on cardiometabolic and anthropometric outcomes.

Eight meta-analyses assessed the effects of flavanone intake and flavanone-rich sources on lipid profile parameters [33,34,35]. Alhabeeb et al. [34] included data on nine RCTs and reported significant reductions in total cholesterol [−9.84 mg/dL (−15.43; −4.24)] and LDL-C [−9.14 mg/dL (−15.79; −2.49)] following orange juice intake (250–750 mL/day for 2–13 weeks). Regarding HDL-C, only Onakpoya et al. [33] reported a significant increase in HDL-C after grapefruit juice intake (MD = 3.21 mg/dL; 95% CI: 0.77 to 5.65). None of the meta-analyses reported significant effects on triglyceride levels following interventions with orange juice (250–750 mL/day) [34], hesperidin supplementation (292–800 mg/day) [35], grapefruit providing 81–119 mg/day of naringin, or grapefruit juice providing 142 mg/day of naringenin [33].

More recently, Huang et al. included 12 randomized controlled trials involving 589 participants and found that hesperidin supplementation improved several cardiometabolic biomarkers, including total cholesterol, LDL-C, fasting blood glucose, CRP, VCAM-1, and ICAM-1, whereas no significant effects were observed for blood pressure, HDL-C, triglycerides, insulin resistance, or anthropometric outcomes [36].

Eight meta-analyses evaluated the effects of flavanone supplementation and flavanone-rich citrus interventions on blood pressure [33,35,37,38]. Hooper et al. [37] reported a significant decrease in diastolic blood pressure after the intake of 0.7 servings/day of citrus fruits for 5 weeks [−3.70 mmHg (−7.02; −0.38)]. In addition, Onakpoya et al. [33] reported a significant decrease in systolic blood pressure after grapefruit juice consumption for 6 to 12 weeks [−2.43 mmHg (−4.77; −0.09)]. No significant results were observed by the other two meta-analyses evaluating hesperidin supplementation and citrus juice interventions [35,38].

Four meta-analyses assessed the effects of flavanones on inflammatory biomarkers including C-reactive protein (CRP) and interleukin-6 (IL-6) [34,39]. Alhabeeb et al. [34] included data from seven studies and reported a significant decrease in CRP plasma concentration after 1 to 12 weeks of supplementation with 250 to 500 mL/day of orange juice [−0.46 mg/L (95% CI: −0.82, −0.12)]. Likewise, Lorzadeh et al. [39] revealed a significant reduction in CRP levels after hesperidin supplementation (500 to 600 mg/day) for 3 to 8 weeks, but only in those RCTs with a parallel design [−0.72 (95% CI: −1.35, −0.09)]. Despite no significant results being reported for IL-6 in these meta-analyses, an RCT by Sánchez Macarro et al. [40] reported a reduction in IL-6 and oxidative stress biomarkers following flavanone intake. Regarding other inflammation-related CVD biomarkers, Lorzadeh et al. [39] reported a significant decrease in vascular cell adhesion molecule-I (VCAM-I) levels after hesperidin supplementation (450–600 mg/day) for 3 to 6 weeks [−22.81 ng/L (95% CI: −44.7, −0.92)]. No significant results were found for the intracellular adhesion molecule-I (ICAM-I) and e-selectin.

Five meta-analyses assessed effects on glucose metabolism biomarkers [33,34,41]. Alhabeeb et al. included five RCTs and found a significant decrease in blood glucose after 4 to 12 weeks of intaking a daily dose of 300 to 500 mL of orange juice [−2.93 mg/dL (95% CI: −5.33, −0.53)] [34]. This study also observed a statistically significant decrease in both insulin [−1.23 μU/mL (−2.08, −0.37)] and the Homeostatic Model Assessment of Insulin Resistance [HOMA-IR (−0.46 (−0.75, −0.18)]. None of the other meta-analyses found significant results.

Five meta-analyses reported the effects of flavanone supplementation on anthropometric outcomes [33,34,42]. Onakpoya et al. [33] included data on three RCTs and showed a significant decrease in waist circumference after a daily intake of ~140 mg of naringenin in the form of grapefruit juice for 6 to 12 weeks compared to the control group [−1.15 cm (95% CI: −1.45; −0.85)]. Conversely, Djafari et al. [42] included two RCTs and reported a significant increase in waist circumference after 500 to 1000 mg/d of hesperidin supplementation for 12 weeks compared with the controls [1.83 (1.03; 2.63)]. Regarding body fat percentage, Onakpoya et al. [33] showed a statistically significant decrease after daily grapefruit juice intake (~140 mg of naringenin) for 6 to 12 weeks compared with the controls [−0.49% (−0.81; −0.18)]. No statistically significant results were found for other anthropometric measurements, including body weight, body mass index (BMI), hip circumference, and lean body mass.

Taken together, findings from interventional studies indicate that flavanones and flavanone-rich citrus interventions may lead to modest improvements in lipid profile, blood pressure, glucose regulation, and inflammatory markers, while effects on anthropometric measures appear limited and heterogeneous.

**Table 2:** Summary of meta-analyses of randomized controlled trials assessing the effects of flavanone intake on cardiometabolic biomarkers and anthropometric outcomes.

Author, Year (Ref)	Studies with Flavanones (n)	Region	Age (y)	Total Participants (n)	Intake	Duration	Heterogeneity (%)	Outcome	Beta (95% CI)
Lipid profile									
Heidari et al., 2025 [36]	14	France, Germany, Iran, Italy, Japan, Netherlands, Spain	18–81	710	Purified flavanone supplement (hesperidin, 292–1000 mg/day)	3–24 weeks	0.0	TC (mg/dL)	−5.82 (−10.56; −1.09)
							0.0	LDL-C (mg/dL)	−4.48 (−7.82; −1.13)
							0.0	HDL-C (mg/dL)	0.67 (−0.70; 2.03)
							68.3	TG (mg/dL)	−13.84 (−26.74; −0.95)
Khorasanian et al., 2023 [43]	13	France, Iran, Italy, Netherlands, Spain	35–73	705	Purified flavanone supplement (hesperidin, 292–1000 mg/day)	3–12 weeks	0.0	TC (mg/dL)	−5.42 (−10.10; −0.75)
							0.0	LDL-C (mg/dL)	−4.31 (−7.58; −1.04)
							0.0	HDL-C (mg/dL)	0.64 (−0.72; 2.00)
							69.5	TG (mg/dL)	−13.85 (−27.21; −0.49)
Huang et al., 2024 [44]	12	France, Iran, Italy, Japan, Netherlands, Spain	21–65	589	Purified flavanone supplement (hesperidin or hesperidin-enriched extracts, 450–1000 mg/day)	3–12	0.0	TC (mmol/L)	−0.20 (−0.31; −0.08)
							0.0	LDL-C (mmol/L)	−0.22 (−0.33; −0.11)
							79.7	TG (mmol/L)	−0.18 (−0.38; 0.01)
							0.0	HDL-C (mmol/L)	0.03 (−0.01; 0.07)
Shylaja et al., 2024 [45]	9	France, Germany, Iran, Italy, Japan, Netherlands, Spain	18–81	2414	Purified flavanone supplement (hesperidin supplementation)	4 to 12 weeks	69	TC	−0.61 (−0.82; −0.41)
							70	LDL-C	−0.55 (−0.94; −0.16)
							12	TG	−0.21 (−0.40; −0.02)
							56	HDL-C	0.04 (−0.25; 0.34)
							60	SBP (mmHg)	−0.29 (−2.21; 1.63)
							49	DBP (mmHg)	0.79 (−0.74; 2.31)
Alhabeeb et al., 2022 [34]	9	Brazil, China, France, UK	>18	414	Flavanone-rich food/beverage (orange juice, 250–750 mL/day)	2 to 13 weeks	0.0	TC (mg/dL)	−9.84 (−15.43; −4.24)
	9						39.6	LDLc (mg/dL)	−9.14 (−15.79; −2.49)
	9						35.7	HDLc (mg/dL)	1.55 (−0.94; 4.04)
	9						0.0	TG (mg/dL)	−2.36 (−11.96; 7.23)
Mohammadi et al., 2019 [35]	8	France, Germany, Iran, Italy, Japan, Netherlands, Spain	18 to 75	476	Purified flavanone supplement (hesperidin, 292–800 mg/day)	4 to 12 weeks	0.0	TC (mg/dL)	−1.04 (−5.65; 3.57)
							6.8	LDLc (mg/dL)	−1.96 (−7.56; 3.64)
							23.2	HDLc (mg/dL)	0.16 (−1.94; 2.28)
							0.0	TG (mg/dL)	0.69 (−5.91; 7.30)
Onakpoya et al., 2017 [33]	2	USA	>18	154	Flavanone-rich food/beverage (grapefruit or grapefruit juice)	6 to 12 weeks	0.0	TC (mg/dL)	−3.02 (−7.01; 0.97)
	2						68.0	LDLc (mg/dL)	−0.71 (−3.69; 2.30)
	3			231			71.0	HDLc (mg/dL)	3.21 (0.77; 5.65)
							0.0	TG (mg/dL)	−1.76 (−8.26; 4.75)
Blood pressure									
Gao et al., 2024 [46]	14	Egypt, England, Iran, Japan, USA, Spain, Italy, France	≥18	656	Purified flavanone supplement (hesperidin, 500–1000 mg/day)	3 to 12 weeks	0.0	SBP (mmHg) and DBP (mmHg)	SBP ( −0.50, 95% CI: −3.25 ~ 2.26), DBP (−0.51, 95% CI: −2.53 ~ 1.51)
Heidari et al., 2025 [36]	14	France, Germany, Iran, Italy, Japan, Netherlands, Spain	18–81	710	Purified flavanone supplement (hesperidin, 292–1000 mg/day)	3–24 weeks	68.0	SBP (mmHg)	−1.84 (−3.51; −0.18)
							34.0	DBP (mmHg)	−0.61 (−1.91; 0.69)
Khorasanian et al., 2023 [43]	13	France, Iran, Italy, Netherlands, Spain	35–73	705	Purified flavanone supplement (hesperidin, 292–1000 mg/day)	3–12 weeks	71.1	SBP (mmHg)	−1.72 (−3.25; −0.18)
							33.6	DBP (mmHg)	−0.51 (−1.75; 0.72)
Huang et al., 2024 [44]	12	France, Iran, Italy, Japan, Netherlands, Spain	21–65	589	Purified flavanone supplement (hesperidin or hesperidin-enriched extracts, 450–1000 mg/day)	3–12 weeks	61.0	SBP (mmHg)	−0.25 (−2.03; 1.53)
							52.0	DBP (mmHg)	−0.55 (−1.78; 0.69)
Shylaja et al., 2024 [45]	9	France, Germany, Iran, Italy, Japan, Netherlands, Spain	18–81	2414	Hesperidin supplementation	4 to 12 weeks	60	SBP (mmHg)	−0.29 (−2.21; 1.63)
							49	DBP (mmHg)	0.79 (−0.74; 2.31)
Hooper et al., 2008 [37]	1	Israel	52 (mean)	12	Flavanone-rich food (citrus fruits, 0.7 servings/day)	5 weeks	-	SBP (mmHg)	−1.80 (−9.97; 6.37)
							-	DBP (mmHg)	−3.70 (−7.02; −0.38)
Mohammadi et al., 2019 [35]	7	England, France, Germany, Iran, Italy, Spain	18 to 81	392	Purified flavanone supplement (hesperidin, 292–800 mg/day)	4 to 12 weeks	54.2	SBP (mmHg)	−0.85 (−3.07; 1.36)
							23.1	DBP (mmHg)	−0.48 (−2.39; 1.42)
Onakpoya et al., 2017 [33]	3	USA	>18	233	Grapefruit or GJ (with 81 to 119 mg/d naringin or 142 mg/d naringenin)	6 to 12 weeks	0.0	SBP (mmHg)	−2.43 (−4.77; −0.09)
								DBP (mmHg)	−1.65 (−3.92; 0.63)
Wang et al., 2021 [38]	3	France, UK	25 to 84	220	Flavanone-rich beverage (orange and grapefruit juice, 340–500 mL/day)	28 to 180 days	0.0	SBP (mmHg)	0.83 (−6.1; 7.81)
Inflammation biomarkers									
Alhabeeb et al., 2022 [34]	7	Brazil, China, France, Italy, UK	>18	338	250 to 500 mL/d OJ	1 to 12 weeks	86.6	CRP (mg/L)	−0.46 (−0.81; −0.12)
Heidari et al., 2025 [36]	14	France, Germany, Iran, Italy, Japan, Netherlands, Spain	18–81	710	Purified flavanone supplement (hesperidin, 292–1000 mg/day)	3–24 weeks	0.0	TNF-α (pg/mL)	−2.74 (−4.58; −0.90)
							59.7	hs-CRP (mg/L)	−0.29 (−0.76; 0.17)
Huang et al., 2024 [44]	12	France, Iran, Italy, Japan, Netherlands, Spain	21–65	589	Purified flavanone supplement (hesperidin or hesperidin-enriched extracts, 450–1000 mg/day)	3–12	0.0	CRP (mg/L)	−0.56 (−1.11; −0.01)
Khorasanian et al., 2023 [43]	13	France, Iran, Italy, Netherlands, Spain	35–73	705	Purified flavanone supplement (hesperidin, 292–1000 mg/day)	3–12 weeks	61.6	CRP (mg/L)	−0.37 (−0.89; 0.15)
							61.0	IL-6 (pg/mL)	−0.28 (−0.89; 0.33)
							0.0	TNF-α (pg/mL)	−2.74 (−4.58; −0.90)
Lorzadeh et al., 2019 [39]	5	France, Iran, Italy	21 to 65	228	Purified flavanone supplement (hesperidin, 292–600 mg/day)	3 to 8 weeks	67.2	CRP (mg/L)	−0.69 (−1.46; 0.08)
									RCTs with parallel design (n = 3): −0.72 (−1.35; −0.09)
	4	France, Iran	30 to 65	204		4 to 8 weeks	6.3	IL-6 (pg/mL)	−0.22 (−0.92; 0.47)
	3	Italy, Iran, Netherlands	18 to 65	167	450 to 600 mg/d hesperidin	3 to 6 weeks	60.9	E-selectin (ng/mL)	−1.89 (−6.07; 2.29)
				116	292 to 500 mg/d hesperidin		0.0	ICAM-1	−7.62 (−16.26; 1.00)
								VCAM-1	−22.81 (−44.7; −0.92)
Glucose metabolism biomarkers									
Onakpoya et al., 2017 [33]	2	USA	>18	162	Grapefruit or GJ (with 81 to 119 mg/d naringin or 142 mg/d naringenin)	6 to 12 weeks	0.0	Blood glucose (mg/dL)	0.10 (−0.04; 0.24)
Heidari et al., 2025 [36]	14	France, Germany, Iran, Italy, Japan, Netherlands, Spain	18–81	710	Purified flavanone supplement (hesperidin, 292–1000 mg/day)	3–24 weeks	37.4	FBS (mg/dL)	−2.92 (−5.66; −0.17)
							83.5	Insulin (µIU/mL)	−0.75 (−2.12; 0.62)
							62.8	HOMA-IR	−0.24 (−0.55; 0.08)
Khorasanian et al., 2023 [43]	13	France, Iran, Italy, Netherlands, Spain	35–73	705	Purified flavanone supplement (hesperidin, 292–1000 mg/day)	3–12 weeks	36.9	FBG (mg/dL)	−2.40 (−5.35; 0.54)
							84.4	Insulin (µIU/mL)	−0.78 (−2.20; 0.64)
							62.7	HOMA-IR	−0.22 (−0.54; 0.10)
Shams-Rad et al., 2020 [41]	6	France, Germany, Iran, Italy, Spain	>18	318	Purified flavanone supplement (hesperidin, 292–582.5 mg/day)	3 to 12 weeks	0.0	Blood glucose (mg/dL)	−1.10 (−3.79; 1.57)
Alhabeeb et al., 2022 [34]	5	Brazil, France	>18	259	300 to 500 mL/d OJ	4 to 12 weeks	0.0	Blood glucose (mg/dL)	−2.93 (−5.33; −0.53)
Alhabeeb et al., 2022 [34]	5	Brazil, France	>18	259	Flavanone-rich food/beverage (orange juice, 300–500 mL/day)	4 to 12 weeks	0.0	Insulin concentration (mg/dL)	−1.23 (−2.08; −0.37)
Alhabeeb et al., 2022 [34]	5	Brazil, UK	>18	249	Flavanone-rich food/beverage (orange juice, 250–500 mL/day)	4 to 12 weeks	0.0	HOMA-IR	−0.46; (−0.75; −0.18)
Huang et al., 2024 [44]	12	France, Iran, Italy, Japan, Netherlands, Spain	21–65	589	Purified flavanone supplement (hesperidin or hesperidin-enriched extracts, 450–1000 mg/day)	3–12	0.0	Fasting blood glucose	−0.15 (−0.29; −0.02)
Anthropometric measurements									
Alhabeeb et al., 2022 [34]	6	Brazil, UK	>18	275	Flavanone-rich food/beverage (orange juice, 250–500 mL/day)	4 to 13 weeks	0.0	BW (kg)	−0.34 (−2.81; 2.14)
	6	Brazil	>18	261	Flavanone-rich food/beverage (orange juice, 300–750 mL/day)	4 to 13 weeks	0.0	BMI (kg/m^2^)	0.39 (−0.50; 1.28)
	5	Brazil, UK	>18	290	Flavanone-rich food/beverage (orange juice, 250–750 mL/day)	8 to 12 weeks	0.0	WC (cm)	0.63 (−0.94; 2.20)
	5	Brazil, UK	>18	203	Flavanone-rich food/beverage (orange juice, 250–500 mL/day)	4 to 13 weeks	0.0	Body fat (%)	0.17 (−0.75; 1.10)
	3	Brazil	20 to 48	170	Flavanone-rich food/beverage (orange juice, 300–500 mL/day)	4 to 12 weeks	0.0	Lean mass (%)	0.25 (−1.45; 1.95)
Khorasanian et al., 2023 [43]	13	France, Iran, Italy, Netherlands, Spain	35–73	705	Purified flavanone supplement (hesperidin, 292–1000 mg/day)	3–12 weeks	0.0	BMI (kg/m^2^)	0.14 (−0.24; 0.53)
							0.0	Weight (kg)	0.36 (0.02; 0.70)
							0.0	WC (cm)	−0.30 (−2.52; 1.92)
Djafari et al., 2021 [42]	2	Iran	45 to 50	164	Purified flavanone supplement (hesperidin, 500–1000 mg/day)	6 to 12 weeks	0.0	BW (kg)	−0.31 (−3.39; 2.36)
	2	Iran	45 to 50	113	Purified flavanone supplement (hesperidin, 500 mg/day)	6 to 12 weeks	73.5	BMI (kg/m^2^)	−0.74 (−2.75; 1.26)
	2	Iran	45 (mean)	149	Purified flavanone supplement (hesperidin, 500–1000 mg/day)	12 weeks	0.0	WC (cm)	1.83 (1.03; 2.63)
	6	Brazil, Iran, UK	>18	277	Purified flavanone supplement (hesperidin, 250–1000 mg/day)	4 to 12 weeks	0.0	BW (kg)	−0.27 (−1.50, 0.96)
	5			251	Purified flavanone supplement (hesperidin, 500–1000 mg/day)		57.6	WC (cm)	−0.54 (−3.66, 2.57)
	7	Brazil, Italy, Iran		308	Purified flavanone supplement (hesperidin, 500–1000 mg/day)		0.0	BMI (kg/m^2^)	0.28 (−0.26; 0.82)
Onakpoya et al., 2017 [33]	3	USA	>18	233	Flavanone-rich food/beverage (grapefruit or grapefruit juice)	6 to 12 weeks	53.0	BW (kg)	−0.45 (−1.06; 0.16)
	2	USA	>18	156			22.0	Body fat (%)	−0.49 (−0.81; −0.18)
	3	USA	>18	233			47.0	WC (cm)	−1.15 (−1.45; −0.85)

BMI, body mass index; BW, body weight; CI, confidence interval; CRP, C-reactive protein; DBP, diastolic blood pressure; GJ, grapefruit juice; HDLc, high-density lipoprotein cholesterol; HOMA-IR, Homeostatic Model Assessment of Insulin Resistance; ICAM-1, intercellular adhesion molecule 1; IL-6, interleukin-6; LDLc, low-density lipoprotein cholesterol; OJ, orange juice; SBP, systolic blood pressure; TC, total cholesterol; TG, triglyceride; USA, United States of America; UK, United Kingdom; VCAM-1, vascular cell adhesion protein 1; WC, waist circumference.

## 4. Discussion

This overview of meta-analyses shows that higher flavanone or flavanone-rich source (e.g., citrus fruit) intake is consistently associated with lower risks of CVD outcomes in prospective cohorts, while interventional studies indicate modest improvements in lipid profile, blood pressure, glucose regulation and inflammatory markers. The beneficial effect of flavanones on cardiometabolic health has been frequently reported. For example, one individual RCT, not included in those meta-analyses, assessed the effects of a daily oral dose of 450 mg of naringenin compared with placebo on Mexican participants with dyslipidemia on different anthropometric and metabolic parameters during 90 days, reporting significant differences in the naringenin group compared to placebo in BMI, total cholesterol, and LDL cholesterol [47]. Another recent RCT [48] reported a significant decrease in high-sensitivity CRP, tumor necrosis factor-alpha (TNF-α) and nuclear factor kappa beta (NFkB) after a daily dose of 1 g of hesperidin supplementation for 12 weeks. However, the potential beneficial effects of flavonoid are not specific to flavanones, as other flavonoid subclasses have also been associated with cardiometabolic benefits, suggesting shared bioactive properties across the flavonoid class. For instance, similar findings have been reported for flavan-3-ols, a distinct flavonoid subclass, with meta-analytic evidence showing inverse associations with cardiometabolic outcomes and improvements in related biomarkers [49].

Recent meta-analyses published between 2023 and 2025 strengthened the evidence supporting hesperidin supplementation. Compared to earlier meta-analyses, these updated analyses included larger numbers of randomized controlled trials and consistently demonstrated modest improvements in total cholesterol, LDL cholesterol, fasting glucose, and systolic blood pressure, while effects on HDL cholesterol, triglycerides, insulin resistance, and anthropometric outcomes remained inconsistent.

The findings should also be interpreted according to the specific flavanone intake evaluated. Dietary flavanones, citrus fruits, citrus juices, purified flavanone supplements (e.g., hesperidin), and individual flavanone compounds (e.g., naringin and naringenin) are not interchangeable sources of flavanone intake. They differ in food matrix, accompanying nutrients, fiber and sugar content, formulation, and bioavailability, all of which may influence their biological effects. Therefore, results from one type of flavanone intake should not be directly extrapolated to another. Furthermore, habitual dietary flavanone intake varies considerably across populations depending on citrus fruit and juice consumption and is generally much lower than the doses evaluated in supplementation trials. Nevertheless, prospective cohort studies included in this umbrella review suggest that higher habitual flavanone intake from natural dietary sources is associated with a lower risk of several cardiometabolic outcomes, supporting the potential importance of dietary flavanones within a healthy dietary pattern. However, evidence from supplementation trials should not be directly extrapolated to habitual dietary flavanone intake, as purified flavanone supplements differ from natural food sources in composition, food matrix, formulation, and bioavailability. Although beneficial cardiometabolic effects have also been reported for other flavonoid subclasses, particularly flavan-3-ols, the present umbrella review focused specifically on flavanones; therefore, a comprehensive comparison across flavonoid subclasses was beyond its scope.

The exact mechanisms underlying the potential effects of flavanones on cardiometabolic disease are not well established yet. Experimental studies indicate that cardiometabolic diseases are associated with oxidative stress, inflammation, dyslipidemia and metabolic function [50], and our overview of meta-analyses suggests that these mechanistic effects would be broadly consistent with the dietary intake of flavanones. Furthermore, they may exert part of their cardiometabolic effects through anti-obesogenic mechanisms by modulating key pathways involved in appetite regulation and food intake, energy expenditure, and intestinal absorption of lipids and carbohydrates [51]. In addition, they may influence adipocyte function by regulating adipogenesis, lipolysis, and β-oxidation, thereby contributing to improved energy balance and reduced fat accumulation [51]. Obesity-related alterations in adipose tissue and ectopic fat accumulation contribute to metabolic dysfunction and are key determinants linking excess adiposity to insulin resistance, type 2 diabetes, and cardiovascular disease. These pathways may therefore represent a plausible mechanism linking flavanone intake to cardiometabolic outcomes [52].

Evidence from cohort studies suggests that higher flavanone intake is associated with modestly smaller increases in body weight, BMI, and waist circumference over time. For instance, in the large European Prospective Investigation into Cancer and Nutrition (EPIC) cohort, Castañeda et al. [53] reported that a higher intake of flavanones was inversely associated with 5 y body weight change. Likewise, in a Mediterranean population, Marranzano et al. [54] observed an inverse association between flavanone intake and excess body weight (BMI ≥ 25) after adjusting for potential confounders [OR: 0.68 (0.48; 0.97)], but not after adjusting for dietary factors (Mediterranean diet score). Adriouch et al. [55] reported results from a French cohort where participants in the higher quartile of intake of flavanones experienced a smaller increase in BMI and a smaller increase in waist circumference after 6 years of follow-up. In a recent cross-sectional analysis from the Fenland Study including 11,568 UK adults, higher flavanone intake was inversely associated with total body fat percentage [β: −0.17 (95% CI: −0.25; −0.09)] and visceral adipose tissue [β: −0.03 cm (−0.05; −0.00)], while no significant associations were observed for subcutaneous abdominal fat thickness or the ratio of visceral adipose tissue (VAT) to subcutaneous adipose tissue (SCAT) [56]. In three large prospective US cohorts including 124,086 participants followed for up to 24 years, flavanone intake showed weaker associations with long-term weight change compared with other flavonoid subclasses. However, after adjustment for citrus juice intake, higher flavanone intake was significantly associated with lower weight gain over 4-year intervals [−0.05 lbs (95% CI: −0.09; −0.01) per additional 25 mg/day] [57].

Moreover, in line with the present study, some recent randomized controlled trials have reported beneficial effects of flavanone-rich interventions on lipid metabolism and oxidative stress biomarkers. These studies have shown reductions in total cholesterol, LDL-C, triglycerides, and oxidized LDL-related markers following supplementation with flavanone. For example, Sánchez Macarro et al. [40] assessed the effect of a combination of polyphenols including 200–300 mg of flavanone glycosides (including naringin, neohesperidin and narirutin) on several biomarkers from healthy Spanish participants. They found a significant decrease in levels of total cholesterol, LDL-c and LDL-oxidase. Likewise, Cheraghpour et al. [48] assessed the effect of 1 g/d of hesperidin for 12 weeks on different lipid metabolism biomarkers from Iranian participants with non-alcoholic fatty liver disease. This study found a significant decrease in levels of total cholesterol and triglycerides. These findings provide additional support for the lipid-modulating effects of flavanones on cardiometabolic diseases.

Furthermore, flavanones may exert part of their cardiometabolic effects through blood pressure lowering mechanisms. A recent clinical study reported that hesperidin-enriched orange juice intake was associated with significant reductions in systolic blood pressure in individuals with pre- and stage 1 hypertension, with greater effects following sustained consumption [58]. Comparable reductions observed with naturally hesperidin-rich orange juice further support a potential blood pressure lowering effect of flavanone-rich interventions.

The cardiometabolic effects of flavanones may also be mediated through additional biological pathways that were not comprehensively captured by the biomarkers evaluated in the included meta-analyses. For example, Barajas-Vegas et al. showed that oral naringenin supplementation significantly increased circulating adiponectin levels in individuals with dyslipidemia [47]. Given the established role of adiponectin in improving insulin sensitivity, reducing inflammation, and protecting against atherosclerotic processes, these findings suggest a potential mechanistic pathway linking flavanone intake to cardiometabolic health [59].

An important consideration when interpreting the intervention evidence is the type of flavanone intake. The included meta-analyses evaluated both flavanone-rich foods and beverages, such as orange juice, grapefruit juice, and citrus fruits, and purified flavanone supplements, primarily hesperidin. Although beneficial effects were reported across both intervention models, purified supplements differ from dietary sources in composition, formulation, and bioavailability. Consequently, findings from supplementation trials should not be directly extrapolated to habitual dietary flavanone intake. Future randomized controlled trials directly comparing flavanone-rich foods with purified flavanone supplements are warranted to clarify their relative effectiveness and clinical relevance.

This overview has several strengths, including a comprehensive synthesis of evidence from both prospective cohort and randomized controlled trials, allowing integration of long-term risk estimates with short-term biomarker responses across multiple cardiometabolic outcomes. By focusing specifically on flavanones as a distinct flavonoid subclass, the study provides a more targeted interpretation of their potential cardiometabolic effects compared with broader flavonoid-based reviews. However, several limitations should be acknowledged. First, the current evidence base remains limited, as several meta-analyses included a relatively small number of primary studies. Second, considerable heterogeneity in study populations, definitions of flavanone intake, intervention characteristics, outcome measures, and adjustment strategies limits the direct comparability of pooled estimates. Furthermore, most cohort meta-analyses did not perform stratified analyses by sex or ethnicity, while evidence from randomized controlled trials was largely derived from relatively small, short-term interventions evaluating surrogate biomarkers rather than clinical cardiometabolic endpoints. Although beneficial effects were observed for several biomarkers, including lipid profile, blood pressure, glucose metabolism, and inflammatory markers, these findings were not entirely consistent across different flavanone sources and formulations and should not be interpreted as definitive evidence of long-term disease prevention. Additionally, several included meta-analyses contained overlapping primary cohort studies and randomized controlled trials, meaning that some original studies may have contributed to more than one pooled estimate. This overlap may have led to the over-representation of certain findings and reduced the independence of the synthesized evidence. Finally, a formal assessment of overlap using a citation matrix and the Corrected Covered Area (CCA) was not performed, which should be considered when interpreting the findings. Therefore, the results of this umbrella review should be interpreted as a qualitative synthesis of the available evidence rather than as definitive quantitative estimates. Another limitation is that we did not perform a formal methodological quality assessment of the included meta-analyses using a standardized tool, such as AMSTAR 2. Consequently, meta-analyses of varying methodological quality were considered in the synthesis, and methodological limitations of individual reviews may have influenced the strength and reliability of the overall conclusions. Therefore, the findings should be interpreted with appropriate caution.

### Practical Implications

The present synthesis of meta-analytic evidence suggests that flavanone intake is associated with more favorable cardiometabolic profiles, particularly in relation to CVD risk, blood pressure, and metabolic biomarkers. These findings may have potential implications for dietary strategies aimed at cardiometabolic disease prevention, particularly through the inclusion of flavanone-rich foods, such as citrus fruits, in habitual diets. The translation of these findings into clinical and dietary practice should consider the bioavailability and safety profile of flavanones. Flavanone glycosides undergo extensive metabolism in the small intestine and liver following hydrolysis by intestinal enzymes, resulting in phase II conjugation and rapid systemic elimination [60]. A substantial proportion escapes small intestinal absorption and is metabolized by the gut microbiota in the colon, generating phenolic metabolites that contribute to systemic exposure [61]. For example, hesperidin is largely metabolized in the colon by the gut microbiota, while its circulating forms are predominantly detected as phase II metabolites, mainly hesperetin sulfate and glucuronide conjugates [62]. In addition, microbiome-derived metabolites such as 3-(4′-hydroxyphenyl)propionic acid, p-coumaric acid, and hippuric acid are among the most commonly identified catabolic products, although marked inter-individual variability in metabolism and excretion has been observed [63]. Overall bioavailability appears to be relatively low and compound-dependent, which may explain inter-individual variability in observed biological effects [64].

For safety, flavanones derived from citrus fruits appear to be safe for human consumption, with no adverse effects reported in available experimental and clinical studies [65]. Animal studies have shown that major dietary flavanones such as hesperidin are well tolerated, non-toxic, and non-cumulative [66,67]. Similarly, human intervention trials have reported good tolerability even at relatively high supplemental doses [48]; for instance, daily administration of up to 1 g of hesperidin for 12 weeks and single doses of naringenin up to 900 mg did not result in clinically relevant adverse events [68]. Collectively, these findings suggest a favorable safety profile for flavanone intake, supporting their potential use in dietary strategies for cardiometabolic health.

## 5. Conclusions

This review indicates that higher flavanone intake is consistently associated with a lower risk of cardiovascular diseases in observational studies. These findings are further supported by interventional evidence showing that flavanones may improve key cardiometabolic risk factors, including total cholesterol, LDL-C, blood pressure, glucose metabolism biomarkers, and C-reactive protein. Taken together, these findings provide an integrated summary of the available evidence, suggesting a potential beneficial role of flavanones in cardiometabolic health while acknowledging the limitations of the underlying literature. These findings may help guide future research toward clarifying optimal intake levels, improving the understanding of flavanone bioavailability, and evaluating their long-term effects in diverse populations, ultimately supporting more informed dietary recommendations.

## Data Availability

No new data were created or analyzed in this study. Data sharing is not applicable to this article.

## Supplementary Materials

The following supporting information can be downloaded at: https://www.mdpi.com/article/10.3390/epidemiologia7040108/s1, File S1: Search strategies used in PubMed; File S2: PRISMA 2020 checklist.

## Abbreviations

BMI	Body Mass Index
CHD	Coronary Heart Disease
CRP	C-Reactive Protein
CVD	Cardiovascular Disease
HDL-C	High-Density Lipoprotein Cholesterol
IL-6	Interleukin-6
LDL-C	Low-Density Lipoprotein Cholesterol
RCT	Randomized Controlled Trial
RR	Relative Risk
SCAT	Subcutaneous Adipose Tissue
TG	Triglycerides
T2D	Type 2 Diabetes
VAT	Visceral Adipose Tissue
WC	Waist Circumference

## References

[1] Eroglu T., Capone F., Schiattarella G.G. (2024). The evolving landscape of cardiometabolic diseases. EBioMedicine.

[2] Chong B., Jayabaskaran J., Jauhari S.M., Chan S.P., Goh R., Kueh M.T.W., Li H., Chin Y.H., Kong G., Anand V.V. (2025). Global burden of cardiovascular diseases: Projections from 2025 to 2050. Eur. J. Prev. Cardiol..

[3] Dong X., Wang J., Wang C., Wang B., Li G., Yang J., Zou T. (2025). Worldwide burden of metabolic risk-related cardiovascular disease from 1990 to 2021, with projections to 2050: A systematic analysis for the Global Burden of Disease Study 2021. Diabetes Obes. Metab..

[4] Zhou L., Nutakor J.A., Larnyo E., Addai-Dansoh S., Cui Y., Gavu A.K., Kissi J. (2024). Exploring socioeconomic status, lifestyle factors, and cardiometabolic disease outcomes in the United States: Insights from a population-based cross-sectional study. BMC Public Health.

[5] Ruskovska T., Maksimova V., Milenkovic D. (2020). Polyphenols in human nutrition: From the in vitro antioxidant capacity to the beneficial effects on cardiometabolic health and related inter-individual variability—An overview and perspective. Br. J. Nutr..

[6] Koch W. (2019). Dietary Polyphenols—Important Non-Nutrients in the Prevention of Chronic Noncommunicable Diseases. A Systematic Review. Nutrients.

[7] Kris-Etherton P.M., Hecker K.D., Bonanome A., Coval S.M., Binkoski A.E., Hilpert K.F., Griel A.E., Etherton T.D. (2002). Bioactive compounds in foods: Their role in the prevention of cardiovascular disease and cancer. Am. J. Med..

[8] Tripoli E., Guardia M.L., Giammanco S., Majo D.D., Giammanco M. (2007). Citrus flavonoids: Molecular structure, biological activity and nutritional properties: A review. Food Chem..

[9] Barreca D., Gattuso G., Bellocco E., Calderaro A., Trombetta D., Smeriglio A., Laganà G., Daglia M., Meneghini S., Nabavi S.M. (2017). Flavanones: Citrus phytochemical with health-promoting properties. BioFactors.

[10] Gandhi G.R., Vasconcelos A.B.S., Wu D.T., Li H.B., Antony P.J., Li H., Geng F., Gurgel R.Q., Narain N., Gan R.Y. (2020). Citrus flavonoids as promising phytochemicals targeting diabetes and related complications: A systematic review of in vitro and in vivo studies. Nutrients.

[11] Azhar S., Sabaha R., Sajjad R., Nadeem F., Amjad A., Hafeez N., Nayab T., Wahid S., Tanweer A. (2022). Effect of Citrus Flavanones on Diabetes; A Systematic Review. Curr. Diabetes Rev..

[12] Stabrauskiene J., Kopustinskiene D.M., Lazauskas R., Bernatoniene J. (2022). Naringin and Naringenin: Their Mechanisms of Action and the Potential Anticancer Activities. Biomedicines.

[13] Benavente-García O., Castillo J. (2008). Update on Uses and Properties of Citrus Flavonoids: New Findings in Anticancer, Cardiovascular, and Anti-inflammatory Activity. J. Agric. Food Chem..

[14] Khan M.K., Zill-E-Huma, Dangles O. (2014). A comprehensive review on flavanones, the major citrus polyphenols. J. Food Compos. Anal..

[15] Gattuso G., Barreca D., Gargiulli C., Leuzzi U., Caristi C. (2007). Flavonoid composition of citrus juices. Molecules.

[16] Addi M., Elbouzidi A., Abid M., Tungmunnithum D., Elamrani A., Hano C. (2021). An overview of bioactive flavonoids from citrus fruits. Appl. Sci..

[17] Zamora-Ros R., Knaze V., Luján-Barroso L., Romieu I., Scalbert A., Slimani N., Hjartåker A., Engeset D., Skeie G., Overvad K. (2013). Differences in dietary intakes, food sources and determinants of total flavonoids between Mediterranean and non-Mediterranean countries participating in the European Prospective Investigation into Cancer and Nutrition (EPIC) study. Br. J. Nutr..

[18] Zamora-Ros R., Knaze V., Luján-Barroso L., Slimani N., Romieu I., Fedirko V., De Magistris M.S., Ericson U., Amiano P., Trichopoulou A. (2011). Estimated dietary intakes of flavonols, flavanones and flavones in the European Prospective Investigation into Cancer and Nutrition (EPIC) 24 hour dietary recall cohort. Br. J. Nutr..

[19] Li T., Zhao Y., Yuan L., Zhang D., Feng Y., Hu H., Hu D., Liu J. (2024). Total dietary flavonoid intake and risk of cardiometabolic diseases: A dose-response meta-analysis of prospective cohort studies. Crit. Rev. Food Sci. Nutr..

[20] Page M.J., McKenzie J.E., Bossuyt P.M., Boutron I., Hoffmann T.C., Mulrow C.D., Shamseer L., Tetzlaff J.M., Akl E.A., Brennan S.E. (2021). The PRISMA 2020 statement: An updated guideline for reporting systematic reviews. BMJ.

[21] Micek A., Godos J., Del Rio D., Galvano F., Grosso G. (2021). Dietary Flavonoids and Cardiovascular Disease: A Comprehensive Dose-Response Meta-Analysis. Mol. Nutr. Food Res..

[22] Aune D., Giovannucci E., Boffetta P., Fadnes L.T., Keum N., Norat T., Greenwood D.C., Riboli E., Vatten L.J., Tonstad S. (2017). Fruit and vegetable intake and the risk of cardiovascular disease, total cancer and all-cause mortality—A systematic review and dose-response meta-analysis of prospective studies. Int. J. Epidemiol..

[23] Kim Y., Je Y. (2017). Flavonoid intake and mortality from cardiovascular disease and all causes: A meta-analysis of prospective cohort studies. Clin. Nutr. ESPEN.

[24] Grosso G., Micek A., Godos J., Pajak A., Sciacca S., Galvano F., Giovannucci E.L. (2017). Dietary Flavonoid and Lignan Intake and Mortality in Prospective Cohort Studies: Systematic Review and Dose-Response Meta-Analysis. Am. J. Epidemiol..

[25] Wang X., Ouyang Y.Y., Liu J., Zhao G. (2014). Flavonoid intake and risk of CVD: A systematic review and meta-analysis of prospective cohort studies. Br. J. Nutr..

[26] Rienks J., Barbaresko J., Nöthlings U. (2017). Association of Polyphenol Biomarkers with Cardiovascular Disease and Mortality Risk: A Systematic Review and Meta-Analysis of Observational Studies. Nutrients.

[27] Zurbau A., Au-Yeung F., Blanco Mejia S., Khan T.A., Vuksan V., Jovanovski E., Leiter L.A., Kendall C.W.C., Jenkins D.J.A., Sievenpiper J.L. (2020). Relation of Different Fruit and Vegetable Sources With Incident Cardiovascular Outcomes: A Systematic Review and Meta-Analysis of Prospective Cohort Studies. J. Am. Heart Assoc..

[28] Li X., Wang C., Yang T., Fan Z., Guo X. (2022). A meta-analysis of prospective cohort studies of flavonoid subclasses and stroke risk. Phytother. Res..

[29] Xu H., Luo J., Huang J., Wen Q. (2018). Flavonoids intake and risk of type 2 diabetes mellitus: A meta-analysis of prospective cohort studies. Medicine.

[30] Guo X.-F., Ruan Y., Li Z.-H., Li D. (2019). Flavonoid subclasses and type 2 diabetes mellitus risk: A meta-analysis of prospective cohort studies. Crit. Rev. Food Sci. Nutr..

[31] Rienks J., Barbaresko J., Oluwagbemigun K., Schmid M., Nöthlings U. (2018). Polyphenol exposure and risk of type 2 diabetes: Dose-response meta-analyses and systematic review of prospective cohort studies. Am. J. Clin. Nutr..

[32] Jia X., Zhong L., Song Y., Hu Y., Wang G., Sun S. (2016). Consumption of citrus and cruciferous vegetables with incident type 2 diabetes mellitus based on a meta-analysis of prospective study. Prim. Care Diabetes.

[33] Onakpoya I., O’Sullivan J., Heneghan C., Thompson M. (2017). The effect of grapefruits (Citrus paradisi) on body weight and cardiovascular risk factors: A systematic review and meta-analysis of randomized clinical trials. Crit. Rev. Food Sci. Nutr..

[34] Alhabeeb H., Sohouli M.H., Lari A., Fatahi S., Shidfar F., Alomar O., Salem H., Al-Badawi I.A., Abu-Zaid A. (2022). Impact of orange juice consumption on cardiovascular disease risk factors: A systematic review and meta-analysis of randomized-controlled trials. Crit. Rev. Food Sci. Nutr..

[35] Mohammadi M., Ramezani-Jolfaie N., Lorzadeh E., Khoshbakht Y., Salehi-Abargouei A. (2019). Hesperidin, a major flavonoid in orange juice, might not affect lipid profile and blood pressure: A systematic review and meta-analysis of randomized controlled clinical trials. Phytother. Res..

[36] Heidari Z., Farahmandpour F., Bazyar H., Pashayee-Khamene F. (2025). Effects of hesperidin supplementation on cardiometabolic markers: A systematic review and meta-analysis of randomized controlled trials. Nutr. Rev..

[37] Hooper L., Kroon P.A., Rimm E.B., Cohn J.S., Harvey I., Le Cornu K.A., Ryder J.J., Hall W.L., Cassidy A. (2008). Flavonoids, flavonoid-rich foods, and cardiovascular risk: A meta-analysis of randomized controlled trials. Am. J. Clin. Nutr..

[38] Wang Y., Gallegos J.L., Haskell-Ramsay C., Lodge J.K. (2021). Effects of chronic consumption of specific fruit (berries, citrus and cherries) on CVD risk factors: A systematic review and meta-analysis of randomised controlled trials. Eur. J. Nutr..

[39] Lorzadeh E., Ramezani-Jolfaie N., Mohammadi M., Khoshbakht Y., Salehi-Abargouei A. (2019). The effect of hesperidin supplementation on inflammatory markers in human adults: A systematic review and meta-analysis of randomized controlled clinical trials. Chem.-Biol. Interact..

[40] Sánchez Macarro M., Martínez Rodríguez J.P., Bernal Morell E., Pérez-Piñero S., Victoria-Montesinos D., García-Muñoz A.M., Cánovas García F., Castillo Sánchez J., López-Román F.J. (2020). Effect of a Combination of Citrus Flavones and Flavanones and Olive Polyphenols for the Reduction of Cardiovascular Disease Risk: An Exploratory Randomized, Double-Blind, Placebo-Controlled Study in Healthy Subjects. Nutrients.

[41] Shams-Rad S., Mohammadi M., Ramezani-Jolfaie N., Zarei S., Mohsenpour M., Salehi-Abargouei A. (2020). Hesperidin supplementation has no effect on blood glucose control: A systematic review and meta-analysis of randomized controlled clinical trials. Br. J. Clin. Pharmacol..

[42] Djafari F., Shahavandi M., Amini M.R., Sheikhhossein F., Shahinfar H., Payandeh N., Jafari A., Djafarian K., Clark C.C.T., Shab-bidar S. (2021). The effects of hesperidin supplementation or orange juice consumption on anthropometric measures in adults: A meta-analysis of randomized controlled clinical trials. Clin. Nutr. ESPEN.

[43] Khorasanian A.S., Fateh S.T., Gholami F., Rasaei N., Gerami H., Khayyatzadeh S.S., Shiraseb F., Asbaghi O. (2023). The effects of hesperidin supplementation on cardiovascular risk factors in adults: A systematic review and dose–response meta-analysis. Front. Nutr..

[44] Huang H., Liao D., He B., Zhou G., Cui Y. (2024). Effects of Citrus flavanone hesperidin extracts or purified hesperidin consumption on risk factors for cardiovascular disease: Evidence from an updated Meta-analysis of randomized controlled trials. Curr. Dev. Nutr..

[45] Shylaja H., Viswanatha G.L., Sunil V., Hussain S.M., Farhana S.A. (2024). Effect of hesperidin on blood pressure and lipid profile: A systematic review and meta-analysis of randomized controlled trials. Phytother. Res..

[46] Gao H., Chen F., Wang S. (2024). Hesperidin reduces systolic blood pressure in diabetic patients and has no effect on blood pressure in healthy individuals: A systematic review and meta-analysis. Phytother. Res..

[47] Barajas-Vega J.L., Raffoul-Orozco A.K., Hernandez-Molina D., Ávila-González A.E., García-Cobian T.A., Rubio-Arellano E.D., Ramirez-Lizardo E.J. (2020). Naringin reduces body weight, plasma lipids and increases adiponectin levels in patients with dyslipidemia. Int. J. Vitam. Nutr. Res..

[48] Cheraghpour M., Imani H., Ommi S., Alavian S.M., Karimi-Shahrbabak E., Hedayati M., Yari Z., Hekmatdoost A. (2019). Hesperidin improves hepatic steatosis, hepatic enzymes, and metabolic and inflammatory parameters in patients with nonalcoholic fatty liver disease: A randomized, placebo-controlled, double-blind clinical trial. Phytother. Res. PTR.

[49] Raman G., Avendano E.E., Chen S., Wang J., Matson J., Gayer B., Novotny J.A., Cassidy A. (2019). Dietary intakes of flavan-3-ols and cardiometabolic health: Systematic review and meta-analysis of randomized trials and prospective cohort studies. Am. J. Clin. Nutr..

[50] Picos-Salas M.A., Cabanillas-Bojórquez L.A., Leyva-López N., Elizalde-Romero C.A., de Jesús Bernal-Millán M., Contreras-Angulo L.A., Lizárraga-Verdugo E.R., Beltrán-Ontiveros S.A., Montoya-Inzunza L.A., Heredia J.B. (2026). Flavanones: Bioavailability and role in modulation of oxidative stress and inflammation in cancer, type-2 diabetes, and cardiovascular diseases. Phytochem. Rev..

[51] Rufino A.T., Costa V.M., Carvalho F., Fernandes E. (2021). Flavonoids as antiobesity agents: A review. Med. Res. Rev..

[52] Chait A., Den Hartigh L.J. (2020). Adipose tissue distribution, inflammation and its metabolic consequences, including diabetes and cardiovascular disease. Front. Cardiovasc. Med..

[53] Castañeda J., Gil-Lespinard M., Almanza-Aguilera E., Llaha F., Gómez J.-H., Bondonno N., Tjønneland A., Overvad K., Katzke V., Schulze M.B. (2023). Association between classes and subclasses of polyphenol intake and 5-year body weight changes in the EPIC-PANACEA study. Obesity.

[54] Marranzano M., Ray S., Godos J., Galvano F. (2018). Association between dietary flavonoids intake and obesity in a cohort of adults living in the Mediterranean area. Int. J. Food Sci. Nutr..

[55] Adriouch S., Kesse-Guyot E., Feuillet T., Touvier M., Olié V., Andreeva V., Hercberg S., Galan P., Fezeu L.K. (2018). Total and specific dietary polyphenol intakes and 6-year anthropometric changes in a middle-aged general population cohort. Int. J. Obes..

[56] Gil-Lespinard M., Forouhi N.G., Imamura F., Zamora-Ros R. (2026). Associations between dietary intake of flavonoids and adiposity: Cross-sectional findings from the Fenland Study, the United Kingdom: Epidemiology and Population Health. Int. J. Obes..

[57] Bertoia M.L., Rimm E.B., Mukamal K.J., Hu F.B., Willett W.C., Cassidy A. (2016). Dietary flavonoid intake and weight maintenance: Three prospective cohorts of 124 086 US men and women followed for up to 24 years. BMJ.

[58] Valls R.M., Pedret A., Calderón-Pérez L., Llauradó E., Pla-Pagà L., Companys J., Moragas A., Martín-Luján F., Ortega Y., Giralt M. (2021). Effects of hesperidin in orange juice on blood and pulse pressures in mildly hypertensive individuals: A randomized controlled trial (Citrus study). Eur. J. Nutr..

[59] Begum M., Choubey M., Tirumalasetty M.B., Arbee S., Mohib M.M., Wahiduzzaman M., Mamun M.A., Uddin M.B., Mohiuddin M.S. (2023). Adiponectin: A promising target for the treatment of diabetes and its complications. Life.

[60] Kay C.D., Pereira-Caro G., Ludwig I.A., Clifford M.N., Crozier A. (2017). Anthocyanins and Flavanones Are More Bioavailable than Previously Perceived: A Review of Recent Evidence. Annu. Rev. Food Sci. Technol..

[61] Pereira-Caro G., Borges G., Van Der Hooft J., Clifford M.N., Del Rio D., Lean M.E.J., Roberts S.A., Kellerhals M.B., Crozier A. (2014). Orange juice (poly)phenols are highly bioavailable in humans. Am. J. Clin. Nutr..

[62] Yang Y., Trevethan M., Wang S., Zhao L. (2022). Beneficial effects of citrus flavanones naringin and naringenin and their food sources on lipid metabolism: An update on bioavailability, pharmacokinetics, and mechanisms. J. Nutr. Biochem..

[63] Williamson G. (2025). Bioavailability of Food Polyphenols: Current State of Knowledge. Annu. Rev. Food Sci. Technol..

[64] Guo X., Li K., Guo A., Li E. (2020). Intestinal absorption and distribution of naringin, hesperidin, and their metabolites in mice. J. Funct. Foods.

[65] Textbook of Natural Medicine-5th Edition. https://www.elsevier.com/books/textbook-of-natural-medicine/pizzorno/978-0-323-43044-9.

[66] Mayumi K., Seiko T., Masa-Aki S., Masao H., Shoji F., Nobuyuki I. (1993). Subchronic toxicity study of methyl hesperidin in mice. Toxicol. Lett..

[67] Damon M., Flandre O., Michel F., Perdrix L., Labrid C., Crastes de Paulet A. (1987). Effect of chronic treatment with a purified flavonoid fraction on inflammatory granuloma in the rat. Study of prostaglandin E2 and F2 alpha and thromboxane B2 release and histological changes. Arzneimittelforschung.

[68] Rebello C.J., Beyl R.A., Lertora J.J.L., Greenway F.L., Ravussin E., Ribnicky D.M., Poulev A., Kennedy B.J., Castro H.F., Campagna S.R. (2020). Safety and Pharmacokinetics of Naringenin: A Randomized, Controlled, Single Ascending Dose, Clinical Trial. Diabetes Obes. Metab..

