# Supplementary material for: Flavanone Intake and Cardiometabolic Outcomes: A Synthesis of Meta-Analytic Evidence from Cohort and Clinical Studies
Source: Epidemiologia (Basel). 2026 Aug 12;7(4):108. doi: 10.3390/epidemiologia7040108 (PMC13512434; doi:10.3390/epidemiologia7040108)
Supplement: Supplementary file 1 [file epidemiologia-07-00108-s001.zip › epidemiologia-4393913-supplementary-File S1.pdf]

Supplementary file S1. Search strategies used in PubMed

```
(
 flavanone*[tiab]
 OR hesperidin[tiab]
 OR hesperetin[tiab]
 OR naringin[tiab]
 OR naringenin[tiab]
 OR eriocitrin[tiab]
 OR eriodictyol[tiab]
 OR (
     citrus[tiab]
     AND
     (
       fruit*[tiab]
       OR juice*[tiab]
       OR orange[tiab]
       OR grapefruit[tiab]
     )
   )
)
AND
(
 cardiovascular[tiab]
 OR cardiometabolic[tiab]
 OR "cardiovascular disease*"[tiab]
 OR "coronary heart disease"[tiab]
 OR stroke[tiab]
```

```
  OR diabetes[tiab]
  OR obesity[tiab]
  OR hypertension[tiab]
  OR "blood pressure"[tiab]
  OR lipid*[tiab]
  OR cholesterol[tiab]
  OR triglyceride*[tiab]
  OR glucose[tiab]
  OR insulin[tiab]
  OR "insulin resistance"[tiab]
  OR HOMA-IR[tiab]
  OR CRP[tiab]
  OR "C-reactive protein"[tiab]
  OR IL-6[tiab]
  OR "interleukin-6"[tiab]
  OR BMI[tiab]
  OR "body weight"[tiab]
  OR "waist circumference"[tiab]
)
AND
(
  Meta-Analysis[pt]
  OR "systematic review"[ti]
  OR "meta-analysis"[ti]
)
NOT
(
  animals[mh] NOT humans[mh]
)
```
